# Higher neutrophil count, higher lymphocyte-to-monocyte ratio, and lower platelet-to-lymphocyte ratio are independently associated with postpartum depression symptoms in twin pregnancies

**DOI:** 10.3389/fimmu.2026.1874901

**Published:** 2026-06-24

**Authors:** Hui Ye, Baorong Gao, Yilan Tian, Fan Yang, Lin Li

**Affiliations:** 1 Department of Obstetrics and Gynecology, West China Second University Hospital, Sichuan University, Chengdu, China; 2 Key Laboratory of Birth Defects and Related Diseases of Women and Children (Sichuan University), Ministry of Education, Chengdu, China

**Keywords:** biomarker, Inflammatory, Neutrophil, postpartum depression, twin

## Abstract

**Introduction:**

Postpartum depression (PPD) is a common complication with adverse maternal and child outcomes. Immune−inflammatory dysregulation has been implicated in its pathogenesis, but evidence from twin pregnancies is limited. This study aimed to investigate associations between routine prenatal inflammatory biomarkers and PPD symptoms in women with twin deliveries.

**Methods:**

This retrospective cohort study included 846 women with twin pregnancies delivered between May 2022 and May 2024 at a tertiary hospital. PPD symptoms were screened within 4 weeks postpartum using the Edinburgh Postnatal Depression Scale. Blood samples at admission provided complete blood counts, from which neutrophil, lymphocyte, monocyte, platelet counts, and derived ratios (NLR, LMR, PLR, SII, SIRI, PIV) were calculated. Multivariable logistic regression was used to assess associations.

**Results:**

Among 846 participants, 145 (17.14%) had PPD symptoms. Women with PPD symptoms had significantly higher neutrophil, lymphocyte, and monocyte counts (all P<0.05). After full adjustment, higher neutrophil count (OR = 1.46, 95% CI: 1.01–2.12, P = 0.047), higher LMR (OR = 1.36, 95% CI: 1.01–1.83, P = 0.041), and lower PLR (OR = 0.98, 95% CI: 0.96–0.99, P = 0.029) were independently associated with PPD symptoms. Lymphocyte count showed a borderline protective effect (OR = 0.33, 95% CI: 0.11–1.02, P = 0.055). Quartile analyses revealed significant dose-response relationships for neutrophils (P for trend <0.001), lymphocytes (P = 0.03), monocytes (P = 0.015), and SIRI (P = 0.023).

**Conclusion:**

Higher neutrophil count, higher LMR, and lower PLR are independently associated with PPD symptoms in twin pregnancies, supporting immune inflammatory involvement in PPD pathophysiology. Future studies should integrate psychosocial and hormonal factors.

## 1 Introduction

Postpartum depression (PPD) affects approximately 10–20% of women worldwide, with higher rates reported in twin pregnancies due to increased physical and psychological burdens (1, 2). PPD not only impairs maternal quality of life but also negatively influences infant development and family functioning (3). Despite its clinical importance, the pathophysiology of PPD remains incompletely understood, and reliable biomarkers for early identification are lacking.

Accumulating evidence suggests that immune-inflammatory dysregulation plays a role in perinatal depression (4–6). Pregnancy induces substantial immunological adaptations, and dysregulation of inflammatory cytokine production is associated with an increased risk of miscarriage and other pregnancy complications (7). Some studies have reported elevated levels of pro-inflammatory markers (e.g., IL-6, IL-8, TNF-α, CRP) in women with PPD symptoms (8). However, most research has focused on single or twin pregnancies without stratification, and the utility of routine, low-cost hematological parameters—such as neutrophil, lymphocyte, monocyte, and platelet counts—remains under-explored.

Derived ratios, including the neutrophil-to-lymphocyte ratio (NLR), lymphocyte-to-monocyte ratio (LMR), platelet-to-lymphocyte ratio (PLR), systemic immune-inflammation index (SII), systemic inflammation response index (SIRI), and pan-immune-inflammation value (PIV), have been proposed as integrative markers of systemic inflammation in various diseases (9, 10). In obstetrics, these indices have been associated with preeclampsia, gestational diabetes, and preterm birth (11–13), but their relationship with PPD—especially in twin gestations—has not been systematically evaluated.

Twin pregnancies represent a unique model of heightened immunological stress, characterized by greater placental mass, higher inflammatory burden, and increased rates of obstetric complications (14). Therefore, examining immune-inflammatory biomarkers in this population may provide clearer insights into the pathways linking inflammation to PPD symptoms. This retrospective cohort study aimed to (1): compare levels of 10 inflammatory biomarkers between women with and without PPD symptoms in twin pregnancies (2); evaluate independent associations after adjusting for potential con-founders; and (3) explore dose-response relationships and subgroup effects.

## 2 Materials and methods

### 2.1 Study design and study population

This retrospective cohort study included all twin deliveries occurring at West China Second University Hospital, Sichuan University—a tertiary academic medical center dedicated to women’s and children’s health—between May 1, 2022, and May 1, 2024. Postpartum depression screening was performed within 4 weeks after delivery using the validated Edinburgh Postnatal Depression Scale (EPDS). Eligibility criteria included (1): maternal age ≥ 18 years (2); ultrasound-confirmed twin pregnancy (3); gestational age at delivery ≥ 28 weeks (4); liveborn infants without congenital malformations (5); no personal or first-degree family history of psychiatric disorders (e.g., major depressive disorder, bipolar disorder, or schizophrenia); and (6) complete, con-temporaneous records of routine admission blood tests and EPDS assessment. Women meeting all six criteria were included. The study protocol was approved by the Institutional Review Board of West China Second University Hospital, Sichuan University (approval number: YXKY2025061), which also granted a waiver of informed consent for this retrospective analysis of anonymized clinical data. All records were de-identified prior to extraction and analysis.

### 2.2 Data collection

Data were extracted from the participants’ medical records, including sociodemographic data such as maternal age, parity, pregnancy weight and height, and hospitalization cost. Pregnancy body mass index (BMI) was calculated by dividing pregnancy weight (kg) by the square of height (m^2^). Information on primiparity, delivery mode, gestational age, blood loss volume and infant congenital malformations was also obtained from medical records. Laboratory data for lymphocyte (×10^9^/L), monocyte (×10^9^/L), neutrophil (×10^9^/L), and platelet count (×10^9^/L) during delivery admission. Inflammatory biomarkers were calculated as follows: NLR = neutrophil count/lymphocyte count, LMR = lymphocyte count/monocyte count, PLR = platelet count/lymphocyte count, SII = platelet count × monocytes/lymphocytes, SIRI = neutrophils × monocytes/lymphocytes, PIV = neutrophil count × platelet count × monocyte count/lymphocyte count.

### 2.3 Postpartum depression symptoms

The primary outcome was postpartum depressive symptoms, assessed using EPDS. The EPDS comprises ten items, each rated on a 4-point scale (0–3), yielding a total score ranging from 0 to 30. Higher scores indicate greater severity of depressive symptoms. The reliability and validity of the Chinese version of the EPDS as a screening tool for postpartum depression have been well established (15). In this study, a score greater than or equal to nine was considered an indicator of postpartum depressive symptoms (16).

### 2.4 Statistical analysis

Nonnormally distributed continuous variables were presented as medians and interquartile ranges (IQRs). Categorical variables were presented as frequencies and percentages. Intergroup comparisons were conducted using Independent samples t-tests, Mann-Whitney U and χ² tests, based on data types. After the concentrations of inflammatory biomarkers were log-transformed, multivariable linear regression was performed to estimate associations between immune-inflammatory concentrations and continuous EPDS scores. Logistic regression models were performed to estimate the association between inflammatory concentrations (both as continuous variables and categorized by quartile distribution) and the dichotomous PPD symptoms variable. Adjusted odds ratios (ORs) and 95% CIs were calculated after adjusting for maternal age, chorionicity, assisted reproductive technology, preterm birth, body mass index, parity, postpartum hemorrhage, gestational diabetes mellitus and hypertensive disorders in pregnancy. Subgroup analyses were conducted, heterogeneity tests were assessed using Cochran’s Q-test, and a P-interaction value was further estimated when heterogeneity was present in each subgroup. As a sensitivity analysis, we also performed multivariable linear regression using the continuous EPDS score as the outcome, adjusting for the same set of covariates. Other analyses with P < 0.05 were considered statistically significant, given their exploratory nature. All statistical analyses were performed using SPSS software (version 25.0).

## 3 Results

### 3.1 Participant characteristics

**Figure 1:**
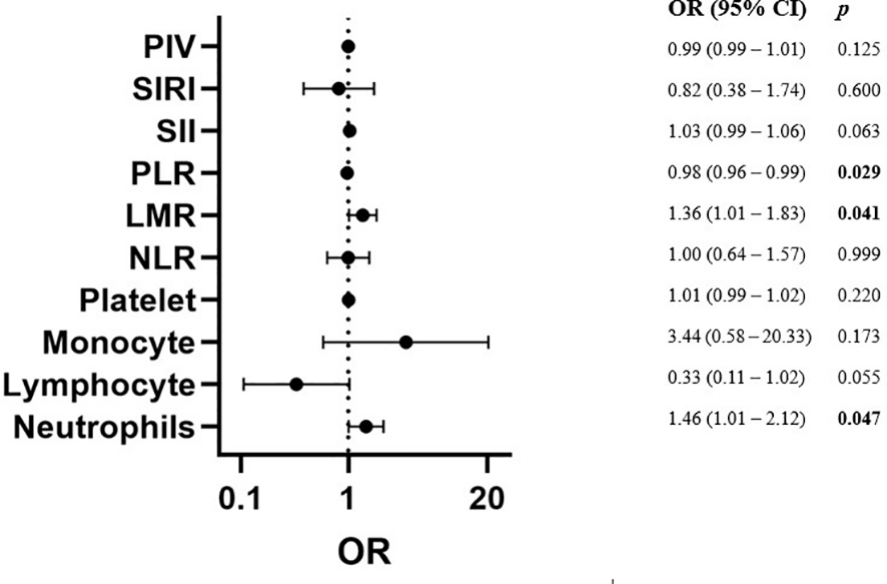
Flowchart of the screening process.

Of 910 women with twin deliveries during the study period, 64 were excluded (10 delivered before 28 weeks, 14 had stillbirth or fetal malformation, 1 had pre-existing anxiety disorder, 39 lacked inflammatory biomarker data). The final analysis included 846 women, of whom 145 (17.14%) had PPD symptoms (EPDS ≥9) and 701 (82.86%) did not (**Figure 1**). The EPDS scores in this study ranged from 0 to 22.

Baseline characteristics are presented in **Table 1**. The median age was 32 years (IQR 29–34 years), and the median body mass index at delivery was 27.24 kg/m² (IQR 25.15 - 28.84 kg/m²). Except for preterm birth (p = 0.020), there were no significant differences between the postpartum depression group and the non-postpartum depression group in terms of age, body mass index, parity, gestational diabetes mellitus, gestational hypertension, assisted reproductive technology, cesarean section, postpartum hemorrhage or chorionicity (all P > 0.05).

**Table 1:** Baseline characteristics of participants.

Characteristics	Overall	PPD symptoms		*p*
		Yes	No	
*n* (%)	846	145 (17.14)	701 (82.86)	–
Age,median (IQR), year	32 (29 –34)	32 (29–34)	32 (29–35)	0.151
BMI at delivery, median (IQR), kg/m2	27.24 (25.15 - 28.84)	27.1 (24.96 - 28.76)	27.29 (25.22-28.84)	0.330
Primiparous, *n* (%)	710 (16.08)	121 (83.45)	589 (84.02)	0.864
Hospitalization cost, meadian (IQR), RMB	16364.59 (14104.64 - 19796.67)	16608.93 (14607.93-21070.75)	16346.9 (14052.84-19456.1)	0.198
Gestational age, median (IQR), week	36 (35 – 37)	36 (35 – 37)	36 (35 – 37)	0.083
Preterm birth, *n* (%)	439 (51.89)	88 (60.69)	351 (50.05)	0.020
GDM, *n* (%)	244 (28.84)	46 (31.72)	198 (28.25)	0.400
HDP, *n* (%)	138 (16.31)	25 (17.24)	113 (16.12)	0.739
ART, *n* (%)	524 (61.94)	84 (57.93)	440 (62.77)	0.275
Cesarean delivery, *n* (%)	829 (97.99)	141 (97.24)	688 (98.15)	0.480
Postpartum hemorrhage, *n* (%)	51 (6.03)	13 (8.97)	38 (5.42)	0.103
Dichorionicity, *n* (%)	642 (75.89)	103 (71.03)	539 (76.89)	0.133

IQR, interquartile ranges. GDM, gestational diabetes mellitus. HDP, hypertensive disorders in pregnancy, ART, assisted reproductive technology. Percentages are calculated column-wise within each group.

### 3.2 Differences in inflammatory biomarkers between groups

**Table 2** presents the median levels of each biomarker. Women with PPD symptoms had significantly higher neutrophil (5.43 vs. 4.96 ×10^9^/L, P = 0.020) and lymphocyte (1.54 vs. 1.47 ×10^9^/L, P = 0.027) counts. PLR was lower in the PPD symptoms group (104.42 vs. 106.98, P = 0.046). No significant differences were observed for monocyte, platelet count, NLR, LMR, SII, SIRI, or PIV (all P > 0.05).

**Table 2:** The differences in inflammatory biomarker levels between the PPD symptoms and non-PPD symptoms.

Immune-inflammatory biomarker	Median (IQR)	PPD symptoms		*p*
		Yes	No	
Neutrophils	5.03 (4.14-6.18)	5.43 (4.41-6.47)	4.96 (4.08-6.12)	**0.020**
Lymphocyte	1.49 (1.23-1.80)	1.54 (1.32-1.87)	1.47 (1.20-1.78)	**0.027**
Monocyte	0.54 (0.43-0.67)	0.58 (0.46-0.73)	0.53 (0.43-0.66)	0.119
Platelet	158 (125–194)	163 (129.50-194.50)	157 (124.50-193.50)	0.190
NLR	3.36 (2.56-4.53)	3.36 (2.63-4.46)	3.36 (2.54-4.54)	0.345
LMR	2.84 (2.17-3.55)	2.84 (2.15-3.46)	2.85 (2.17-3.57)	0.807
PLR	106.47 (79.47-141.73)	104.42 (77.06-126.96)	106.98 (79.70-144.19)	**0.046**
SII	54.96 (39.84-79.40)	56.23 (41.92-79.39)	54.54 (39.23-79.40)	0.642
SIRI	1.80 (1.22-2.62)	1.93 (1.38-2.82)	1.77 (1.19-2.57)	0.714
PIV	279.02 (177.37-443.84)	300.29 (204.43-469.21)	276.22 (174.79-436.91)	0.516

Bold values indicate statistical significance (p < 0.05).

### 3.3 Quartile analysis and dose-response relationships

After adjusting for maternal age, chorionicity, assisted reproductive technology, preterm birth, body mass index, parity, postpartum hemorrhage, gestational diabetes mellitus and hypertensive disorders in pregnancy, significant trends across quartiles were observed for neutrophils, lymphocytes, monocytes, and SIRI (**Table 3**). For neutrophils, com-pared with Q1, the OR for Q4 was 2.613 (95% CI: 1.501–4.550, P < 0.001) with a strong linear trend (P for trend <0.001). For lymphocytes, Q4 had an OR of 2.029 (95% CI: 1.173–3.511, P = 0.011; trend P = 0.03). For monocytes, only Q4 reached significance (OR = 1.86, 95% CI: 1.138–3.042, P = 0.013; trend P = 0.015). For SIRI, Q4 showed an OR of 1.905 (95% CI: 1.108–3.275, P = 0.020; trend P = 0.023). No significant trends were found for platelet, NLR, LMR, PLR, SII, or PIV (all trend P > 0.05).

**Table 3:** Associations between maternal immune-inflammatory biomarkers during pregnancy and the postpartum period with PPD symptoms.

Immune-inflammatory biomarker	Quartile	OR	95% CI	*P*
Neutrophil				
	Quartile 1	Ref		
	Quartile 2	1.60	0.90-2.84	0.112
	Quartile 3	2.04	1.12-3.58	**0.013**
	Quartile 4	2.61	1.50-4.55	**<0.001**
*p* for trend		**<0.001**		
Lymphocyte				
	Quartile 1	Ref		
	Quartile 2	1.85	1.07-3.22	**0.029**
	Quartile 3	1.60	0.92-2.80	0.099
	Quartile 4	2.03	1.17-3.51	**0.011**
*p* for trend		**0.030**		
Monocyte				
	Quartile 1	Ref		
	Quartile 2	0.94	0.55-1.61	0.823
	Quartile 3	0.95	0.56-1.63	0.857
	Quartile 4	1.86	1.14-3.04	**0.013**
*p* for trend		**0.015**		
Platelet				
	Quartile 1	Ref		
	Quartile 2	1.14	0.67-1.92	0.632
	Quartile 3	1.32	0.80-2.20	0.287
	Quartile 4	1.18	0.70-1.10	0.539
*p* for trend		0.442		
NLR				
	Quartile 1	Ref		
	Quartile 2	1.47	0.87-2.47	0.151
	Quartile 3	1.44	0.85-2.42	0.172
	Quartile 4	1.16	0.67-1.99	0.602
*p* for trend		0.641		
LMR				
	Quartile 1	Ref		
	Quartile 2	0.96	0.58-1.60	0.880
	Quartile 3	1.04	0.63-1.72	0.871
	Quartile 4	0.83	0.49-1.39	0.476
*p* for trend		0.570		
PLR				
	Quartile 1	Ref		
	Quartile 2	0.97	0.58-1.60	0.891
	Quartile 3	1.12	0.69-1.84	0.644
	Quartile 4	0.74	0.43-1.26	0.266
*p* for trend		0.420		
SII				
	Quartile 1	Ref		
	Quartile 2	1.32	0.78-2.24	0.301
	Quartile 3	1.37	0.81-2.31	0.242
	Quartile 4	1.28	0.75-2.18	0.369
*p* for trend		0.381		
SIRI				
	Quartile 1	Ref		
	Quartile 2	1.57	0.91-2.73	0.108
	Quartile 3	1.70	0.98-2.91	0.057
	Quartile 4	1.91	1.11-3.28	**0.020**
*p* for trend		**0.023**		
PIV				
	Quartile 1	Ref		
	Quartile 2	1.13	0.66-1.94	0.659
	Quartile 3	1.49	0.88-2.52	0.135
	Quartile 4	1.45	0.86-2.44	0.169
*p* for trend		0.104		

Associations were adjusted for maternal age, chorionicity, assisted reproductive technology, preterm birth, body mass index, parity, postpartum hemorrhage, gestational diabetes mellitus and hypertensive disorders in pregnancy. PPD, postpartum depression. OR, odd ratios. CI, confidence interval. Bold values indicate statistical significance (p < 0.05).

### 3.4 Association between inflammatory biomarkers and PPD symptoms

**Figure 2:**
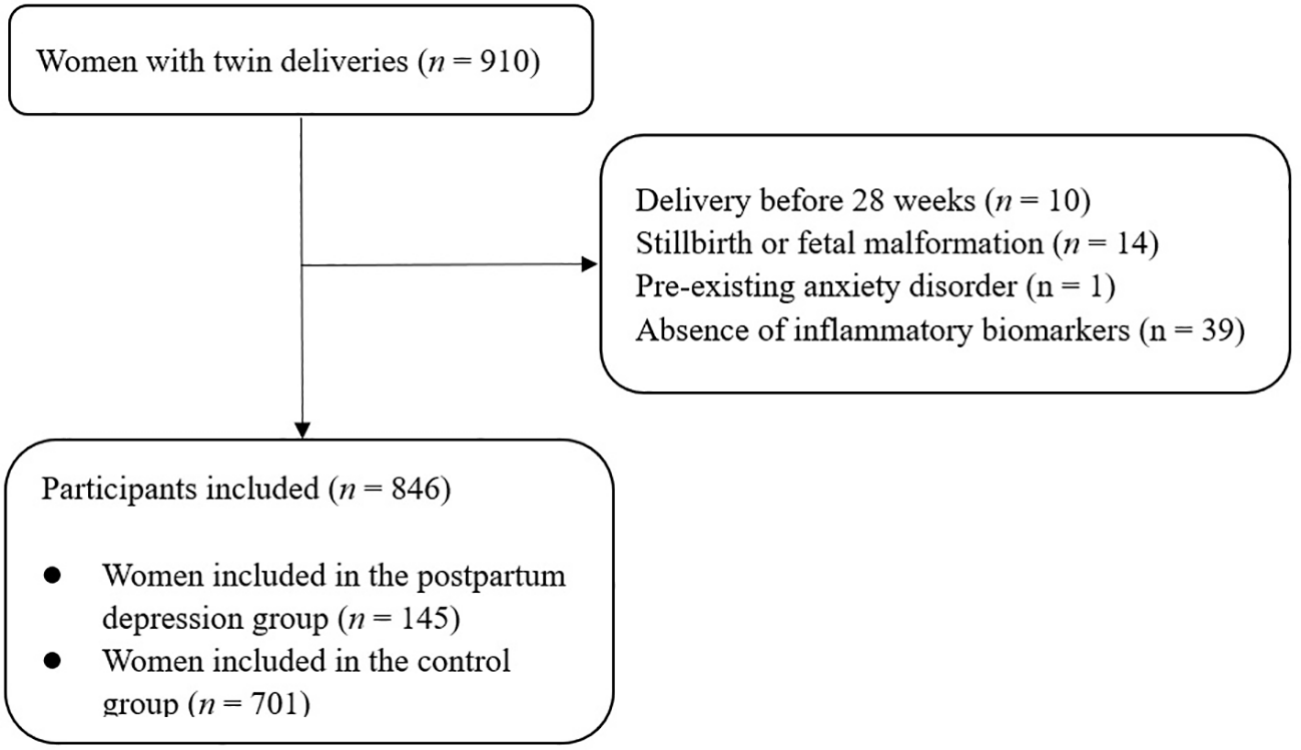
Forest plots illustrating the association between prenatal immune-inflammatory biomarkers with postpartum depression symptoms. Black yuan represent OR values, and black horizontal lines indicate the corresponding 95% confidence intervals. Association was evaluated using logistic regression model, adjusting for maternal age, BMI, parity, preterm birth, postpartum hemorrhage, chorionicity, GDM, HDP and ART. OR, odd ratio; BMI, body mass index; GDM, gestational diabetes mellitus; HDP, hypertensive disorders of pregnancy; ART, assisted reproductive technology. Full regression results including all covariates are provided in **Supplementary Table 1**.

**Figure 2** shows the results of multivariable logistic regression with each biomarker entered as a continuous variable, adjusting for maternal age, chorionicity, assisted reproductive technology, preterm birth, body mass index, parity, postpartum hemorrhage, gestational diabetes mellitus and hypertensive disorders in pregnancy. Full regression results including all covariates are provided in **Supplementary Table 1**. Three biomarkers reached statistical significance:

Neutrophil count (per 10^9^/L increase): OR = 1.46 (95% CI: 1.01–2.12, P = 0.047).

LMR (per unit increase): OR = 1.36 (95% CI: 1.01–1.83, P = 0.041).

PLR (per unit increase): OR = 0.98 (95% CI: 0.96–0.99, P = 0.029).

Lymphocyte count showed a borderline protective association (OR = 0.33, 95% CI: 0.11–1.02, P = 0.055). SII exhibited a marginal positive trend (OR = 1.03, 95% CI: 0.99–1.06, P = 0.063). No significant associations were observed for monocyte, platelet, NLR, SIRI, or PIV.

In sensitivity analyses using the continuous EPDS score, none of the inflammatory biomarkers showed a statistically significant linear association (all P > 0.05; **Supplementary Table 2**), which may reflect the non−linear dose−response patterns observed in the quartile analyses (**Table 3**).

### 3.5 Subgroup analyses

Subgroup analyses for neutrophils, PLR, and LMR are shown in **Table 4**. The association of neutrophils with PPD symptoms was significantly stronger in primiparous women (OR = 1.164, 95% CI: 1.052–1.289) than in multiparous women (OR = 0.798, 95% CI: 0.586–1.086), with a significant interaction (P = 0.039). For PLR, the protective effect was more evident in women with preterm birth (OR = 0.99, 95% CI: 0.98–1.00) compared with those without preterm birth (OR = 1.00, 95% CI: 0.99–1.01); interaction P = 0.039. For LMR, a significant interaction was observed with HDP (P = 0.023): higher LMR was associated with lower PPD symptoms odds in women with HDP (OR = 0.59, 95% CI: 0.35–0.98), but not in those without HDP. No other significant interactions were detected.

**Table 4:** Subgroup analyses of neutrophils, PLR, and LMR during the postpartum period in relation to PPD symptoms.

Characteristics		*n*	Neutrophils		PLR		LMR	
			OR (95%CI)	p*	OR (95%CI)	p*	OR (95%CI)	p*
Age								
	<35	635	**1.15 (1.02-1.28)**	0.564	0.99 (0.98 - 1.01)	0.134	0.95 (0.78 - 1.12)	0.394
	≥35	211	1.05 (0.89-1.23)		0.99 (0.98 - 1.01)		1.24 (0.96 - 1.60)	
BMI								
	<25	192	1.14 (0.96 - 1.36)	0.832	**0.99 (0.98 - 1.00)**	0.206	1.11 (0.86 - 1.43)	0.421
	≥25	654	1.11 (0.99 - 1.23)		0.99 (0.98 - 1.01)		0.97 (0.81 - 1.15)	
Parity								
	Primiparous	710	**1.16 (1.05 - 1.29)**	**0.039**	0.99 (0.98 - 1.01)	0.317	0.97 (0.82 - 1.15)	0.340
	Multiparous	136	0.80 (0.59 - 1.09)		0.99 (0.98 - 1.01)		1.19 (0.85 - 1.66)	
Preterm birth								
	Yes	439	1.06 (0.94 - 1.19)	0.172	**0.99 (0.98 - 1.00)**	**0.039**	1.11 (0.90 - 1.37)	0.216
	No	407	**1.21 (1.04 - 1.40)**		1.00 (0.99 - 1.01)		0.93 (0.74 - 1.17)	
GDM								
	Yes	244	1.18 (0.99 - 1.40)	0.263	0.99 (0.98 - 1.01)	0.598	0.96 (0.71 - 1.29)	0.323
	No	602	1.08 (0.97 - 1.20)		1.00 (0.99 - 1.01)		1.06 (0.90 - 1.25)	
HDP								
	Yes	138	**1.35 (1.04 - 1.74)**	0.33	1.00 (0.99 - 1.01)	0.483	**0.59 (0.35 - 0.98)**	**0.023**
	No	708	1.08 (0.97 - 1.19)		1.00 (0.99 - 1.01)		1.10 (0.94 - 1.28)	
ART								
	Yes	524	**1.17 (1.02 - 1.34)**	0.161	1.00 (0.99 - 1.01)	0.231	0.93 (0.74 - 1.16)	0.381
	No	322	1.05 (0.93 - 1.20)		0.99 (0.98 - 1.01)		1.10 (0.90 - 1.34)	
Chorionicity								
	Dichorionicy	642	1.07 (0.97 - 1.19)	0.196	1.00 (0.99 - 1.01)	0.581	0.96 (0.81 - 1.14)	0.221
	Monochoronicity	204	**1.29 (1.05 - 1.59)**		1.00 (0.99 - 1.01)		1.16 (0.86 - 1.55)	

*p for the test of heterogeneity.

Associations were adjusted for maternal age, chorionicity, assisted reproductive technology, preterm birth, body mass index, parity, postpartum hemorrhage, gestational diabetes mellitus and hypertensive disorders in pregnancy. PPD, postpartum depression. OR, odd ratios. CI, confidence interval. Bold values indicate statistical significance (p < 0.05).

## 4 Discussion

To our knowledge, this is the first study to comprehensively evaluate 10 inflammatory biomarkers in relation to PPD symptoms specifically in twin pregnancies. This retrospective cohort study of 846 women with twin pregnancies systematically evaluated the associations between ten routine immune-inflammatory biomarkers and postpartum depression symptoms. The main findings are (1): higher neutrophil count, higher LMR, and lower PLR were independently associated with increased odds of PPD symptoms after com-prehensive adjustment; and (2) these associations showed dose-response patterns in quartile analyses. Collectively, our results support the involvement of immune-inflammatory mechanisms in PPD symptoms but argue against the use of these parameters as standalone clinical prediction tools.

Neutrophils are first-line responders in innate immunity and their elevation reflects systemic low-grade inflammation. In our study, women with PPD symptoms had significantly higher neutrophil counts than controls, and each 1×10^9^/L increase conferred a 46% higher risk. This finding is consistent with several previous reports linking elevated neutrophil counts to perinatal depression (17, 18). Mechanistically, inflammatory cytokines such as IL-6 and TNF-α, often co-elevated with neutrophils, activate indoleam-ine-2,3-dioxygenase (IDO), shifting tryptophan metabolism from serotonin synthesis to kynurenine pathway (19). The resulting neuroactive metabolites (e.g., quinolinic acid) can trigger depressive behavior (20). Furthermore, neutrophil-derived myeloperoxidase and reactive oxygen species may contribute to oxidative stress, which has been implicated in depression (21, 22). The dose-response relationship we observed (P for trend <0.001) adds credibility to a causal interpretation, although longitudinal studies are needed.

LMR is calculated as lymphocyte count divided by monocyte count. In our multi-variable model, higher LMR was associated with increased the risk of PPD symptoms (OR = 1.36). This seems counterintuitive because lymphocytes are generally considered protective against inflammation. However, the quartile analysis for lymphocytes showed that both Q2 and Q4 had elevated ORs (1.85 and 2.03), suggesting a non-linear or U-shaped relationship. Meanwhile, monocytes showed a significant association only in the highest quartile (OR = 1.86). The independent effect of LMR may be driven by the relative pre-dominance of monocytes over lymphocytes. Monocytes are key producers of pro-inflammatory cytokines (IL-1β, IL-6, TNF-α) and can migrate to the brain, contributing to neuroinflammation (23, 24). Thus, a higher LMR might reflect a state where monocyte activity is not sufficiently counterbalanced by lymphocytes. Alternatively, the borderline protective effect of lymphocytes (OR = 0.33, P = 0.055) suggests that lymphopenia could be a risk factor; LMR increases when lymphocytes decrease, which could explain the positive association. Future studies should examine absolute cell counts alongside ratios to disentangle these effects.

PLR showed a negative association with PPD symptoms (OR = 0.98 per unit increase). Although the effect size is small, the interquartile range of PLR is range from 79.47 to 141.73, so a 2% reduction per unit translates to a clinically meaningful difference between extremes. Platelets store and release serotonin, brain-derived neurotrophic factor, and other neuroactive substances (25). Higher PLR may indicate better platelet functional reserve or a relative increase in platelet count that buffers against mood disturbances. Conversely, lower PLR—often seen in inflammatory conditions due to platelet consumption or lymphocytosis—might reflect ongoing systemic inflammation. Our finding contrasts with some studies reporting elevated PLR in depression (26), but those studies were mostly in non-perinatal populations. The unique immune milieu of pregnancy and the postpartum period may alter the direction of these associations.

Several established inflammatory indices (NLR, SII, SIRI, PIV) were not significantly associated with PPD symptoms in our adjusted models. This may be because these composite indices incorporate NLR, which itself showed no association (OR = 1.00). In twin pregnancies, the baseline inflammatory state is already elevated, potentially diluting the signal from ratios that are more sensitive in healthy populations. Additionally, the strong correlation between neutrophil count and these indices could lead to collinearity issues when all are considered separately. Our finding that the simple neutrophil count outperformed complex ratios suggests that in high-risk populations like twin gestations, individual cell counts may be more informative than derived indices. Consistent with a large recent study by Xie et al. (2025) involving 17,500 postpartum women, we observed that lower lymphocyte count showed a borderline protective effect against PPD symptoms. However, unlike Xie et al., we found no significant associations for NLR or SII, and we identified higher LMR and lower PLR as independent correlates of PPD symptoms. These differences may reflect the unique inflammatory milieu of twin pregnancies, which have a higher baseline immune burden than singleton gestations (6a).

Our subgroup analyses identified three significant interactions. The neutrophil-PPD symptoms association was stronger in primiparous women, suggesting that first-time mothers may exhibit a more reactive innate immune response to twin pregnancy. The protective effect of PLR was limited to preterm deliveries, possibly because preterm birth involves greater inflammation where platelet-related mechanisms become relevant. LMR was protective only in women with HDP, indicating that this ratio may reflect a beneficial immunological profile under high-inflammatory conditions. These exploratory findings require confirmation in larger, prospective studies.

Our findings have two main implications. First, they reinforce the role of immune-inflammatory pathways in PPD symptoms, suggesting that interventions targeting inflammation (e.g., omega-3 fatty acids, anti-inflammatory diets, or even IL-6 antagonists) could be explored for PPD prevention in high-risk groups. Second, we attempted to use inflammatory markers as risk predictors for PPD, but the modest effect sizes and low discriminative ability (AUC ≈ 0.62) clearly indicate that these routine biomarkers cannot replace psychosocial screening (e.g., EPDS) for clinical decision-making. They should be viewed as adjunctive biological correlates rather than predictive tools.

Several limitations should be acknowledged (1). Retrospective design: although data were collected prospectively, we cannot establish causality. The direction of association remains uncertain; inflammation may cause PPD symptoms, or PPD-related behavioral changes (e.g., poor sleep, altered diet) could influence immune parameters (2). Lack of psychosocial confounders: we did not measure social support, marital conflict, sleep quality, or birth trauma, all of which are strong PPD predictors. Women who experienced more labor-related stress or had lower prenatal support between childbirth and EPDS completion, and these unmeasured confounders could bias our estimates. Future studies should integrate biological markers with detailed psychosocial assessments. Residual confounding is likely (3). Single time point for biomarkers: blood was drawn at admission for delivery, which may not reflect the trajectory of inflammation across pregnancy or the postpartum period (4). Twin-specific population: results may not generalize to singleton pregnancies (5). Multiple testing: we performed numerous comparisons without correction (e.g., Bonferroni), increasing the risk of type I errors. Thus, significant findings should be considered hypothesis-generating (6). EPDS as a screening tool: a score ≥9 indicates probable PPD symptoms but not a clinical diagnosis; misclassification may have occurred.

## 5 Conclusions

In this large retrospective cohort of twin pregnancies, higher neutrophil count, higher LMR, and lower PLR were independently associated with postpartum depression symptoms, supporting the involvement of immune-inflammatory mechanisms in PPD symptoms. These associations exhibited dose-response patterns and were more pronounced in primiparous women and those with HDP. However, the modest effect sizes and low discriminative accuracy indicate that routine blood-derived inflammatory biomarkers alone are insufficient for clinical risk prediction. Future prospective studies should integrate biological, psychological, and social factors to develop clinically useful prediction models and to elucidate causal pathways.

## Data Availability

The original contributions presented in the study are included in the article/**Supplementary Material**. Further inquiries can be directed to the corresponding author/s.
