# Supplementary material for: Higher neutrophil count, higher lymphocyte-to-monocyte ratio, and lower platelet-to-lymphocyte ratio are independently associated with postpartum depression symptoms in twin pregnancies
Source: Front Immunol. 2026 Jun 24;17:1874901. doi: 10.3389/fimmu.2026.1874901 (PMC13343226; doi:10.3389/fimmu.2026.1874901)
Supplement: Supplementary file 1 [file Table1.docx]

|  | P value | OR | 95% CI | |
| --- | --- | --- | --- | --- |
| Maternal age | 0.284 | 0.974 | 0.927 | 1.022 |
| BMI | 0.32 | 0.968 | 0.909 | 1.032 |
| Preterm birth | 0.108 | 1.389 | 0.93 | 2.074 |
| GDM | 0.431 | 1.177 | 0.785 | 1.765 |
| HDP | 0.79 | 1.07 | 0.651 | 1.758 |
| ART | 0.496 | 0.86 | 0.558 | 1.326 |
| Postpartum hemorrhage | 0.102 | 1.772 | 0.893 | 3.517 |
| Dichorionicity | 0.507 | 0.848 | 0.521 | 1.38 |
| Parimiparous | 0.962 | 1.013 | 0.606 | 1.693 |
| Neutrophils | 0.047 | 1.461 | 1.005 | 2.124 |
| Lymphocyte | 0.055 | 0.329 | 0.106 | 1.023 |
| Monocyte | 0.173 | 3.441 | 0.583 | 20.327 |
| Platelet | 0.22 | 1.008 | 0.995 | 1.021 |
| NLR | 0.999 | 1 | 0.637 | 1.57 |
| LMR | 0.041 | 1.361 | 1.012 | 1.831 |
| PLR | 0.029 | 0.98 | 0.963 | 0.998 |
| SII | 0.063 | 1.029 | 0.998 | 1.06 |
| SIRI | 0.6 | 0.816 | 0.383 | 1.742 |
| PIV | 0.125 | 0.998 | 0.994 | 1.001 |

Table S1. Full multivariable logistic regression results including all covariates (adjusted ORs, 95% CIs, and P values for each of the nine confounders) for the ten biomarkers.
