# Supplementary material for: Higher neutrophil count, higher lymphocyte-to-monocyte ratio, and lower platelet-to-lymphocyte ratio are independently associated with postpartum depression symptoms in twin pregnancies
Source: Front Immunol. 2026 Jun 24;17:1874901. doi: 10.3389/fimmu.2026.1874901 (PMC13343226; doi:10.3389/fimmu.2026.1874901)
Supplement: Supplementary file 2 [file Table2.docx]

Table S2. Associations between inflammatory biomarkers and continuous EPDS score: multivariable linear regression

|  | Unstandardized B | Std. Error | Beta | t | Sig | 95% CI | |
| --- | --- | --- | --- | --- | --- | --- | --- |
| Neutrophils | 0.121 | 0.165 | 0.072 | 0.729 | 0.466 | -0.204 | 0.445 |
| Lymphocyte | 0.325 | 0.576 | 0.065 | 0.564 | 0.573 | -0.806 | 1.456 |
| Monocyte | -0.734 | 0.823 | -0.089 | -0.893 | 0.372 | -2.349 | 0.88 |
| Platelet | 0.001 | 0.007 | 0.024 | 0.221 | 0.825 | -0.011 | 0.014 |
| NLR | 0.01 | 0.226 | 0.007 | 0.045 | 0.964 | -0.433 | 0.453 |
| LMR | -0.061 | 0.174 | -0.024 | -0.35 | 0.726 | -0.403 | 0.281 |
| PLR | -0.006 | 0.008 | -0.09 | -0.672 | 0.502 | -0.022 | 0.011 |
| SII | 0.008 | 0.011 | 0.098 | 0.768 | 0.443 | -0.013 | 0.03 |
| SIRI | 0.161 | 0.324 | 0.107 | 0.497 | 0.62 | -0.476 | 0.798 |
| PIV | -0.001 | 0.001 | -0.159 | -0.837 | 0.403 | -0.004 | 0.001 |
